# Regulation of dendritic cell biology by amino acids and their transporters

**DOI:** 10.3389/fimmu.2025.1626973

**Published:** 2025-07-04

**Authors:** Shanlin Chen, Gongwei Li, Zihao Jiang, Yuekang Xu, Adila Aipire, Jinyao Li

**Affiliations:** Xinjiang Key Laboratory of Biological Resources and Genetic Engineering, College of Life Science & Technology, Xinjiang University, Urumqi, China

**Keywords:** amino acids, amino acids transporters, dendritic cells, immune response, metabolic reprogramming

## Abstract

Dendritic cells (DCs) play a central role in inducing both immunity and tolerance as specialized antigen-presenting cells (APCs). The immunometabolic firestorm in recent years has focused our attention on how DCs use energy and respond to nutritional changes that affect immune functions. Like in every other cell, such metabolic events as the concentration of free amino acids, membrane-bound transporter proteins, key metabolic enzymes, and sensors (e.g., mTOR and GCN2), also profoundly affect the function and fate of DCs. Therefore, dysregulation of amino acid metabolism can cause metabolic reprogramming of DCs, leading to or accelerating the occurrence of various immunological disorders, like type 1 diabetes, rheumatoid arthritis, and cancer. Since amino acids cannot directly enter the cell to participate in metabolic activities, their transporters act as critical metabolic gatekeepers. To catch up with the rapid development in the immune metabolism field, this review summarized recent studies on the potential roles of different amino acids and their transporters in the regulation of DCs biology to offer new insights for immune-dysregulated diseases and explore new therapeutic targets.

## Introduction

**Figure 1:**
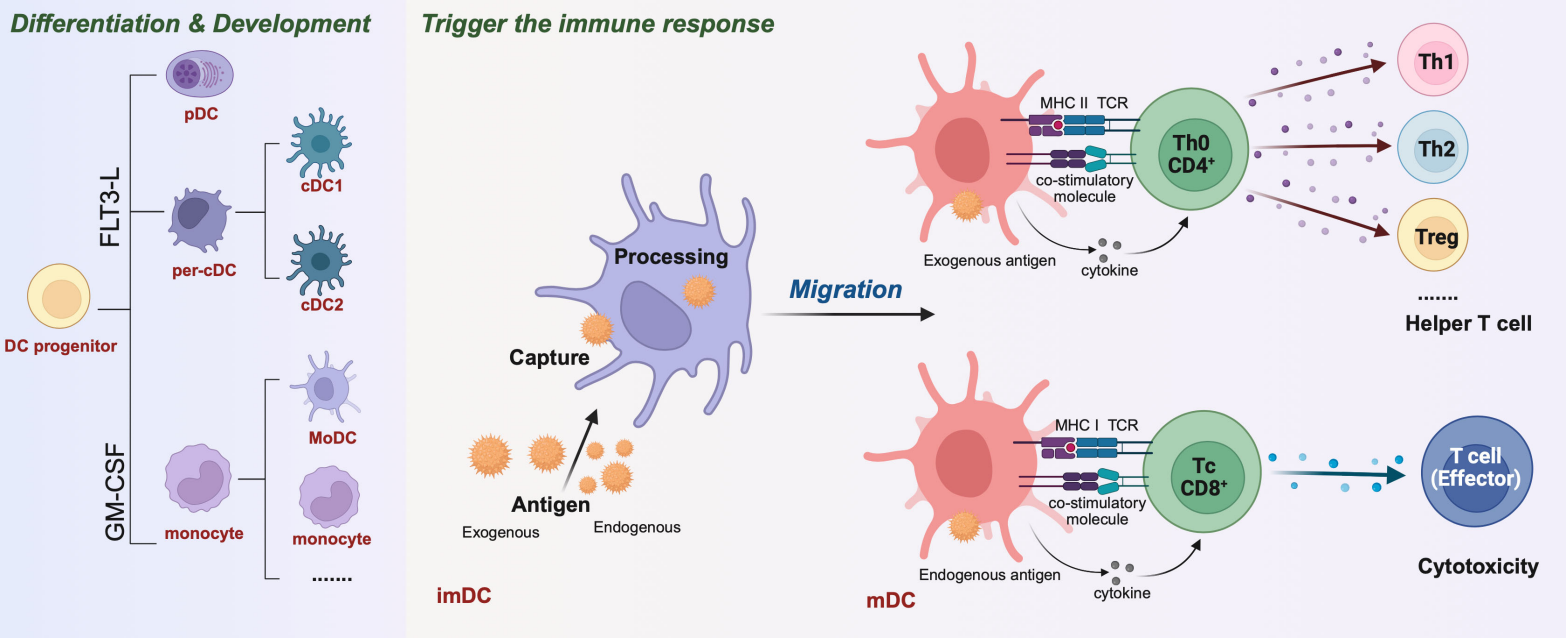
Differentiation & development of dendritic cells (DCs) and activation of T cells to initiate adaptive immune responses. The different DCs are derived from a common DC precursor that requires the transcription factor FMS-like tyrosine kinase 3 ligand (FLT3-L) for development and expression, such as plasma DC (pDC) and Per-cDC. Others can differentiate under induction by GM-CSF, IL-4 into mononuclear DCs(MoDC), monocyte and other. Initial immature dendritic cells (imDC) capture exogenous and endogenous antigens and present them to the cell surface after “treatment” to mature dendritic cells (mDC). Among them, the exogenous antigen mDC was processed. Through the MHC II-TCR complex, costimulatory molecules and secretory factors, CD4^+^Th0 cells were activated. Th0 cells were regulated by cytokines and differentiated into Th1, Th2, Treg and other helper T cells. mDCs that handle endogenous antigens activate CD8^+^Tc cells through MHC I-TCR complexes and costimulatory molecules. These molecules are regulated by cytokines and differentiate into effector T cells, mediating cytotoxic immunity.

Dendritic cells (DCs), as the most effective antigen-presenting cell (APC), is the bridge between innate immunity and adaptive immunity (1). At the resting state, the majority of DCs *in vivo* are in an immature state with strong antigen capture and processing capacity but weaker ability to activate T cells. While stimulated by antigen, the DCs become mature, phagocytose the antigen, and migrate to secondary lymphoid organs, delivering the antigen to T cells to initiate adaptive immunity. DCs comprise multiple subsets with distinct functions but can be broadly classified into two major subsets, myeloid DCs [including classical/conventional DCs (cDCs), which are further subdivided into classical type 1 DCs (cDC1s) and classical type 2 DCs (cDC2s), monocyte-derived DCs (MoDCs), and Langerhans cells] and plasmacytoid DCs (pDCs) (2, 3). Two cytokines, fms-related tyrosine kinase 3 ligand (Flt3-L) and granulocyte-macrophage colony-stimulating factor (GM-CSF), are thought to be essential regulators of DCs development *in vivo*: Flt3-L supports the development of cDCs and pDCs derived from bone marrow (BM) precursors, whereas GM-CSF contributes to the development of MoDCs, as well as inflammation-induced myeloid DCs (4)(**Figure 1**). As powerful APCs, DCs play a crucial role in promoting inflammation and immunosurveillance on the one hand, and suppressing inflammation, and enabling immune escape on the other. The different immune functions performed by DCs, as well as the transition from an immature to a mature state in response to stimulus signals, are modulated by multiple factors, such as selective gene expression, cytokines stimulation, antigen presentation, nutrient metabolism and so on (5, 6). Among these, the metabolic reprogramming of immune cells has received increasing attention, but metabolic changes in DCs are still poorly understood.

The role of amino acids in protein synthesis is well-defined, and a large number of studies in recent years have demonstrated that amino acid metabolism has a profound effect on various types of immune cells, including bone marrow cells and lymphocytes (7). Throughout evolution, the catabolic pathways of amino acids have evolved to be primary regulatory nodes in the control of immune responses. For example, when T cells compete with cancer cells for amino acid supply in the tumor microenvironment (TME), they increase leucine uptake through antigenic signaling mediated by the interaction of the TCR and the L-type amino acid transporter (LAT1) (8). Branched-chain amino acids (BCAAs) are catabolized by branched-chain amino transferases (BCATs) to produce the metabolite β-hydroxy-β-methylbutyric acid (HMB), which facilitates the conversion of T helper 1 (Th1)-type cells to T helper 2 (Th2)-type cells (9). In addition, the concentration of extracellular L-arginine controls the expression of T-cell antigen receptor ζ-chain (CD3ζ) to influence the antigen recognition function of T cells (10). Several amino acids, such as leucine, glutamine, and glutamate, have been shown to regulate tumor-associated B cells (11–13). Given the important role of DCs in the immune response and controlling of tumor growth, this has prompted us to revisit how metabolic reprogramming of DCs, particularly amino acid metabolism, affects the function and fate of DCs. Like in every other cell, amino acids in DCs are also important for fueling mitochondrial respiration, for protein synthesis, as well as acting as a source of carbon and nitrogen for the synthesis of various other macromolecules. In addition to responses to intracellular metabolic changes, DCs also have a sensitive perception of amino acids in the extracellular environment, dynamically adjusting their own state to adapt to (patho)physiological needs (14). For example, Kakazu et al. found that plasma amino acid imbalance of advanced cirrhosis (ACM) suppresses the maturation of DCs. They cultured MoDCs *in vitro* using serum-free culture medium consistent with the average concentration of the plasma amino acid from a healthy volunteer (HCM) and that from patients with ACM and compared DCs function and glucose-amino acid metabolism in each medium. The results demonstrated that imbalanced amino acid levels in ACM interfered with the mitochondrial metabolism of immature DCs, causing a reduction in ATP levels and an increase in glucose consumption, increased aerobic glycolysis in immature DCs under ACM could not be further increased by LPS stimulation, because glycolysis was already promoted before adding LPS. This resulted in lower expression of the maturation-associated surface molecules CD83 and CD86, as well as the cytokine IL-12, by DCs under ACM than under HCM. However, this could be enhanced by supplementation with BCAAs, especially L-valine and L-leucine (15). This indicates that several aspects of DCs biology, including differentiation, activation, and core metabolism, are highly sensitive to changes in amino acid intake and concentration in the environment. In addition to the stimulation of DCs by the amino acids in the environment, the transporters of amino acids on the surface of DCs can also sense and transmit amino acid signals. Mechanistic target of rapamycin complex 1 (mTORC1) is a forward regulator of amino acids, and the amino acids transferred into the cell first activate mTORC1, triggering downstream growth and synthesis-related events. By coupling the mTORC1 and general control nonderepressible 2 (GCN2) signaling pathways, downstream effector factors are activated to participate in the growth, differentiation, and immune function of DCs. The GCN2 signaling pathway can timely sense the deficiency of amino acids and maintain homeostasis by downregulating protein synthesis levels, inducing amino acids transporters (AATs) expression, and upregulating the expression of amino acid biosynthesis-related genes through downstream effectors (16).

Amino acid metabolism in DCs profoundly influences immune responses and DC-T cells cross-talk in the immune microenvironment. There are three sources of amino acids required for cell acquisition: one is from external intake, such as amino acids produced by the breakdown and metabolism of proteins or peptides in feed, which is the major way for animal cells to obtain amino acids; the second source is the reuse of amino acids produced by the breakdown of proteins in the body’s own tissues, such as amino acids formed by the degradation of extracellular matrix proteins; the third origin comes from the amino acids formed in the body through amino acid conversion. Due to the polar nature of amino acids, they cannot function freely through the cell membrane and therefore require the assistance of protein transporters on the cell membrane to complete transport in and out of the cell. In recent years, many studies have focused on the metabolic reprogramming of DCs, comprehensively exploring the roles of various nutrients, including amino acids, in the immunological functions of DCs.

**Figure 2:**
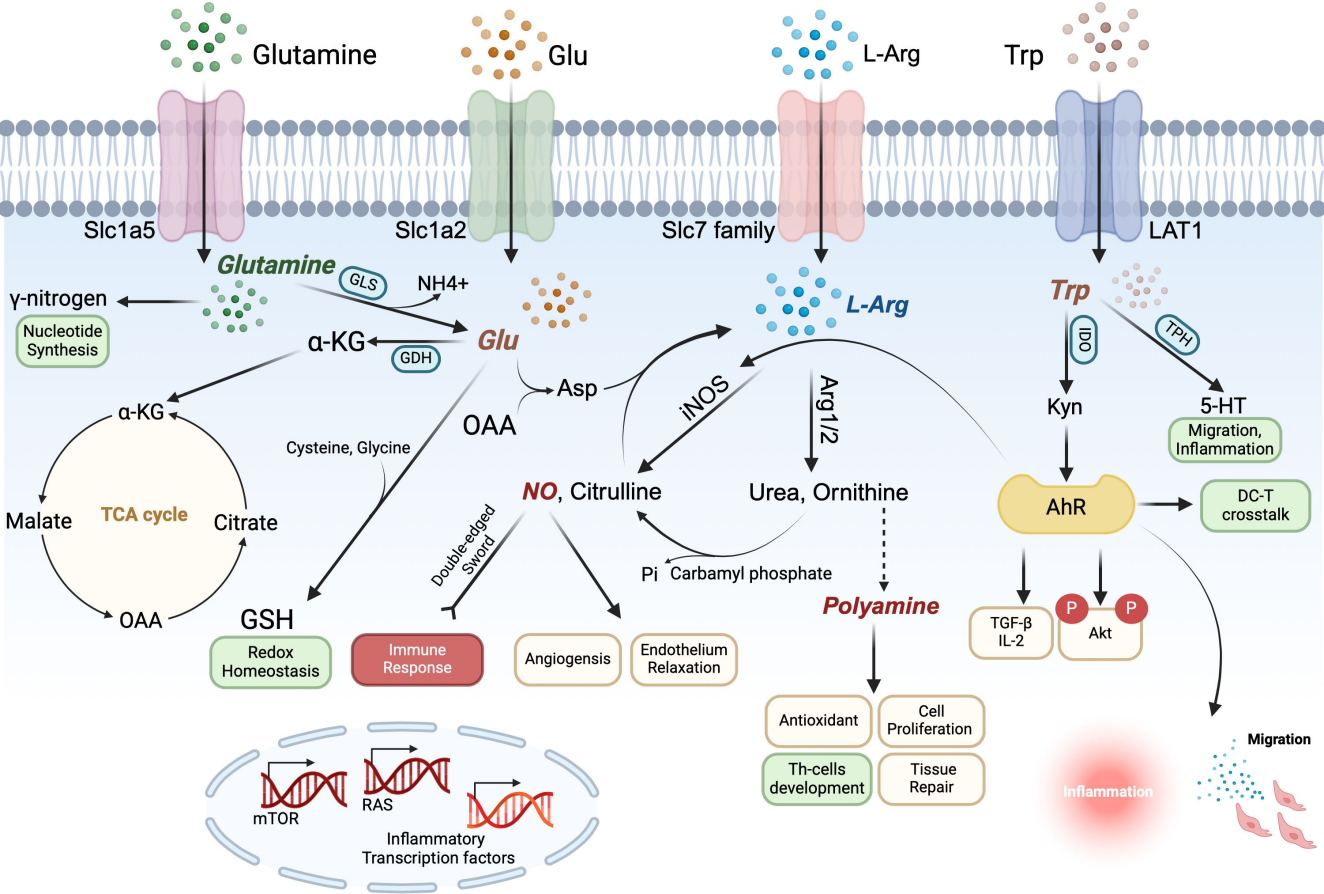
Arginine (Arg), tryptophan (Trp), Glutamine (Gln) and glutamate (Glu) form an amino acid metabolic network that synergistically regulates dendritic cells (DCs) immune function. Gln enters the cytosol via Slc1a5, where it contributes its γ-nitrogen to synthesize nucleic acids and hexosamine and is converted to Glu in the mitochondria via a deamination reaction catalyzed by glutaminase (GLS); Glu is converted by glutamate dehydrogenase (GDH) to α-ketoglutarate (α-KG), an intermediate of the TCA cycle, which is used to generate ATP for the cellular energy needs. In the cytoplasm, Glu interacts with cysteine and glycine to produce glutathione (GSH) to maintain redox homeostasis. Glu also generates aspartic acid (Asp) in the mitochondria from oxaloacetate (OAA), an intermediate product of the TCA cycle, which is catalyzed by argininosuccinate synthetase and argininosuccinate lyase to further generate Arg to enter the urea cycle. Arg transported intracellularly by the Slc7 family and Arg of Glu origin can generate NO and polyamines in the presence of Arg1/2 and iNOS, both of which perform several biological functions in the DCs. In particular, NO can positively or negatively regulate the immune response, playing the role of a double-edged sword, and polyamines can regulate the differentiation and development of T cells. Trp is transported into the cell by LAT1 and catalyzed by IDO and TPH to generate Kyn and 5HT, respectively. Kyn can act on AhR to promote Treg-related cytokine secretion and inhibit activation of downstream inflammation-related pathways, maintaining the tolerogenic phenotype of DCs and also affecting DC-T crosstalk, while 5HT may also be involved in inflammatory activation and migration events.

**Figure 3:**
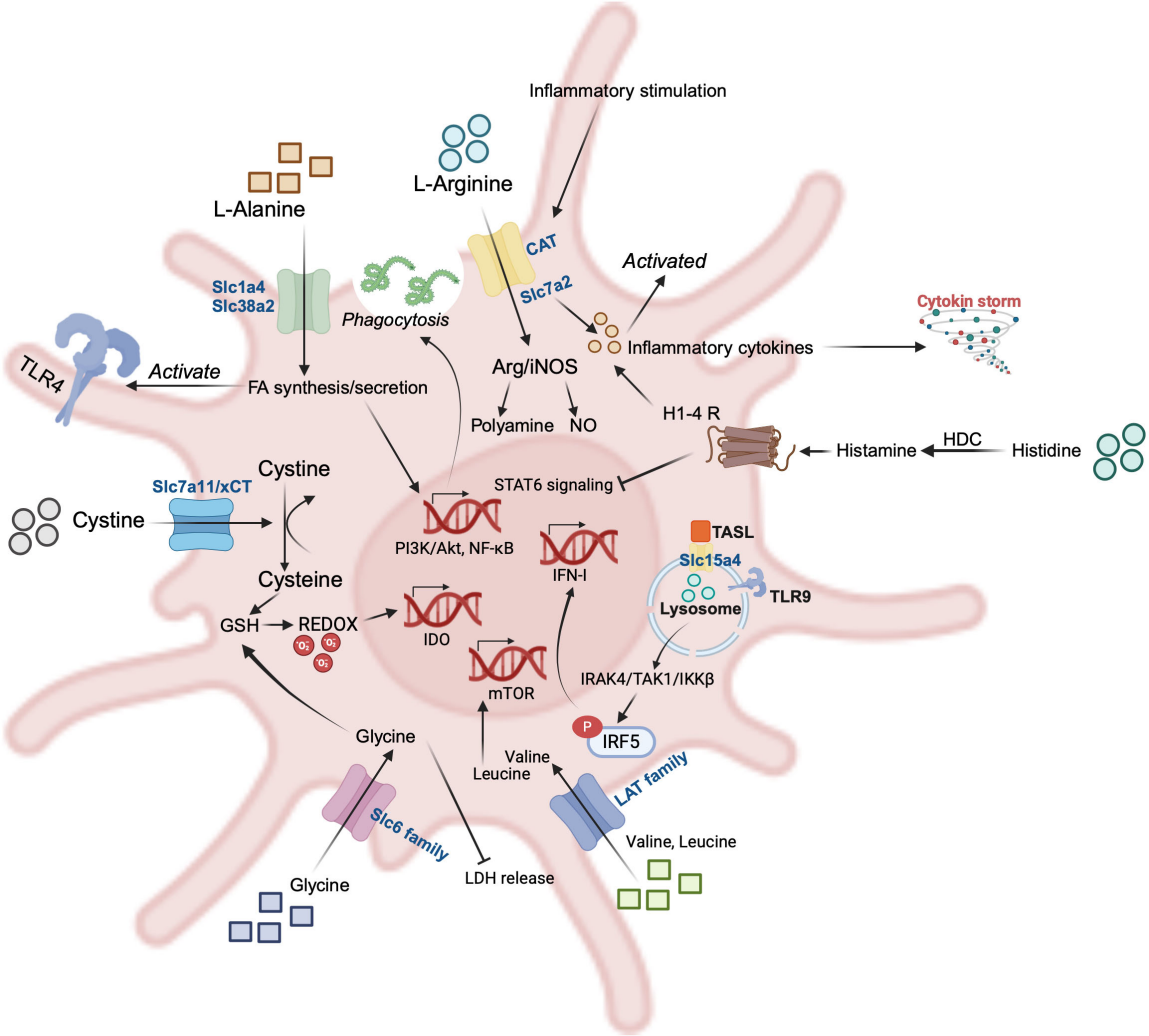
Representative amino acids (AAs) and their transporters in dendritic cells (DCs). L-alanine (Ala) promotes phagocytosis of pathogens, and its translocation to the intracellular compartment via Slc1a4 and Slc38a2 enhances FA biosynthesis and secretion, and activation of the PI3K/Akt and NF-κB pathways further activates TLR-4 signaling; The CAT family of arginine (Arg) transporters is activated in response to inflammatory stimuli, which promotes secretion of inflammation-associated cytokines; histidine (His) is catalyzed by histidine decarboxylase (HDC) to produce histamine, which acts on the histamine receptor on the surface of DCs and is able to inhibit the STAT6 signaling pathway while promoting the secretion of inflammatory cytokines; the lysosomal surface has Slc15a4, a transporter of His, whose interactions with the adaptor TASL are necessary for the downstream phosphorylation of IRF5; Branched-chain amino acids (BCAAs) such as Valine and Leucine are transported intracellularly by the LAT family, which activates mTOR, a classical pathway for amino acid perception; Glycine and cystine are transported intracellularly by the Slc6 family and xCT to participate in glutathione (GSH) synthesis and maintain redox homeostasis.

In this review, we focus on the potential roles of amino acids and transporter-mediated amino acid metabolism in the regulation of DC immunobiology. We provide an overview of recent studies on various amino acids and their transporters (e.g. Arg, Trp, Glu….) (**Figures 2**, **3**, **Tables 1**, **2**), which are critically important in regulating DCs homeostasis and function in various disease models. A deeper comprehension of the roles of amino acids and their transporters in DCs may provide new insights into cancer immunotherapy and other immune-related clinical applications.

**Table 1:** Metabolism and function of various amino acids in DCs.

Amino acid	Metabolic pathways	Metabolic intermediates	Comment	Ref.
Alanine	Biosynthesis of fatty acid	Palmitate	L-Alanine triggers a cascade response in the TLR4 pathway and initiate an adaptive immune response.	(88, 89)
Arginine	Urea cycle	Polyamine, NO	Arginine promotes DCs endocytosis of its own RNA through the urea cycle. DCs can regulate the metabolism of arginine to obtain tolerance and exert immunosuppressive effects.	(23)
Cystine	Biosynthesis of glutathione	Cysteine	Inside the cell, cystine is rapidly reduced to cysteine and functions both as an intracellular antioxidant and as the rate-limiting precursor for the biosynthesis of the major cellular antioxidant glutathione.	(94)
Histidine	Decarboxylation of histidine	Histamine	Histidine is catalyzed to decarboxylate into histamine, playing a crucial role in infection, inflammation, and tumorigenesis.	(165–167)
Glycine	Gluconeogenesis, Biosynthesis of glutathione	Glutathione	Glycine can exert immunomodulatory effects by affecting various inflammation-related signaling pathways. It can serve as an antioxidant to provide protection for DCs.	(168)
Valine	Decomposition of BCAA	Acetyl-CoA, succinyl-CoA	BCAA, especially valine, modulated MoDCs allostimulatory capacity and cytokine production. The depletion of extracellular valine inhibits DCs maturation.	(108)
Leucine	mTORC1/LXRα signaling pathway	Acetyl-CoA, succinyl-CoA	Leucine depletion reduces DCs function and inhibits mTOR signaling.	(108)
Phenylalanine	Oxidative deamination	α-Ketoglutaric acid, tyrosine	The oxidative deamination of phenylalanine affects the antigen presentation of DCs and its cross-talk with T-lymphocytes. IL4I1-mediated deamination of phenylalanine inhibit the proliferation of CD3-stimulated T lymphocytes.	(119)
Tryptophan	Kyn pathway, 5-HT pathway, indole pathway	Kynurenine	Tryptophan can rescue the overactivation of DCs immune function through IDO, 3-HAA, and other metabolic processes, thereby inhibiting T cell responses.	(54)
Glutamate	Tricarboxylic acid cycle	α-Ketoglutaric	Glutamate is an important carbon and nitrogen source in the metabolism of DCs, and a key component in the synthesis of antioxidant-reduced glutathione (GSH).	(65, 94)

**Table 2:** The mechanism and function of amino acid transporters in DCs.

Gene	Alias	Transport mechanism	Substrate	Comment	Ref.
SLC7A11	xCT	Xc–, sodium-independent, anionic amino acid transport system	Cystine, glutamate	SLC7A11 modulates the intracellular cysteine content and glutamate efflux from DCs and acts as brake on DCs efferocytosis.	(70, 146)
SLC38A2	ATA2, Sat2, Snat2	Enables neutral L-amino acid: sodium symporter activity	Alanine	Tumor cells and cDC1s compete for glutamine uptake via the transporter SLC38A2 to tune anti-tumor immunity.	(84)
SLC7A2	Atrc2, Cat2	Cationic amino acid transporter, y+ system	Arginine	SLC7A2 is highly expressed in DCs and actively participates in their activation and anti-inflammatory response.	(128)
SLC15A4	PTR4,PHT1	Enables peptide: proton symporter activity	Histidine	SLC15A4 is a novel link between inflammasome activation and phagocytosis, promoting inflammasome activity via mTORC1 signaling and autophagy restraint in DCs.	(141, 142)
SLC1A2	Eaat2, GLT1	XAG–, sodium- and potassium-dependent transporter	Glutamate	SLC1A2 participates in multiple processes such as L-glutamate transmembrane translocation and glutathione biosynthesis.	(143, 144)

## Amino acids of the regulation of DC maturation and function

In this section we first discuss how amino acids support the metabolic reprogramming critical for DCs activation, focusing on arginine, tryptophan, and glutamate, which are actively involved in immune metabolism (**Figure 2**). Next, we describe the impact of microbiological events involving some other amino acids (Alanine, Cystine, Histidine, Valine, etc.) on DCs biosynthesis and effector molecule production (**Figure 3**).

### Arginine

Arginine is a building block for protein synthesis. In addition, it also serves as a substrate for distinct metabolic pathways (such as the urea cycle) that profoundly affect immune cell biology, especially DCs, macrophages, and T cells. A growing body of evidences indicates that, over the course of the evolution of the immune system, arginine has been selected as a node for the regulation of immune responses (17).

There are two key enzymes involved in arginine metabolism: arginase (ARG) and nitric oxide synthase (NOS). While ARG is able to catabolize arginine into ornithine and polyamines to promote cell proliferation and tissue repair, NOS breaks down arginine into NO to be involved in immune response and inflammation regulation (18, 19). The balance between the activities of ARG and NOS determines the direction of arginine metabolism, which in turn affects the function of the DCs (20, 21). For example, the polyamines produced by the urea cycle catalyzed by arginase can cause nucleic acid aggregation and promote the internalization of its RNA. Psoriasis is a chronic autoimmune disease in which auto-nucleic acids from keratinocytes activate TLR receptors in DCs endosomes, initiating an autoimmune inflammatory cascade (22). Arginine-derived polyamines protect psoriatic keratinocyte-released auto-RNA from degradation and promote endocytosis of auto-RNA by DCs, which promotes TLR7-dependent RNA sensitization and IL-6 production, exacerbating disease progression (23). A previous study also showed that intervention with ARG inhibitors upregulated the expression of surface co-stimulatory molecules and cytokine secretion in DCs (24). According to this, Wirla et al. drove BMDCs into a tolerance profile by inhibiting ARG by a mechanism that may be related to increasing co-stimulatory molecules on effector T cells and stimulating Treg cell production (24). Arg1 activity in DCs has also been correlated with the ability to regulate the differentiation of peripheral helper T cells. The Th2 cytokines, such as IL-4 and IL-10, represent efficient inducers of Arg1 expression, whereas IFN-γ induces iNOS and inhibits Arg1 on DCs (24, 25). Given that arginine depletion causes numerous immunosuppressive effects in immune cells (e.g. macrophages, DCs), depletion of arginine is a recognized strategy that pathogens use to evade immune effector mechanisms. ARG and arginine deiminases (ADI) have been associated with pathogen virulence, both deplete arginine, but their reaction products and immunomodulation differ. Using ADI to stimulate human monocyte-derived DCs, it was found that the depletion of arginine caused by ADI significantly increased the expression of TNF-α and inhibited the secretion of IL-10 and IL-12p40, as well as the upregulation of surface molecules CD83 and CD86. In addition, the deficiency of arginine can also inhibit the activation of the mTOR signaling pathway, indicating that ADI by arginine depletion had immunomodulatory effects on DCs (26). Overall, Arg1 expression may compromise antimicrobial defenses *in vivo* during pathogen infection through three distinct mechanisms: 1) Arg1 depletes L-arginine from DCs, thereby preventing NOS2 from generating NO, which is effective in defending the organism against pathogen infections; 2) Arg1 from DCs competes for L-arginine required by T cells in the immune microenvironment; 3) Arg1-catalyzed generation of polyamines is essential for the growth of various parasites (27). In addition, polyamines can weaken the immune response by inhibiting T-cell proliferation and cytokine secretion (28, 29).

Another important pathway of arginine metabolism is the generation of NO catalyzed by NOS, which was previously defined as a critical regulator of tumor immunity. The quantity of NO within the immune microenvironment directly influences the metabolic states and functions of various immune cells. NOS facilitates the production of NO and citrulline from arginine and is the main source of NO in DCs. NO can regulate survival of inflammatory DCs (30), DCs migration (31), inflammatory cytokine production (32), T cell activation and proliferation (33). NO can mediate both host-protective and host-toxic effects by interfering with pathogen replication and/or inhibiting host mitochondrial respiration, leading to the metabolic remodeling of DCs (34). The relevance of arginine metabolism in DCs is further highlighted by the identification of a specific DCs subpopulation that is characterized by TNF-α and NOS2 expression, hence known as TNF-α, iNOS-producing DCs (Tip-DCs) (35, 36) Tip-DCs exert pro-inflammatory roles conferring resistance to *Listeria monocytogenes*, *Brucella melitensis*, and *Leishmania* major infections (36–38). In addition to pathogen resistance, the pro-inflammatory activity of Tip-DCs and their interaction with tumor-specific CD8^+^T cells also lead to excellent anti-tumor responses (39). On the contrary, the NO produced by it can inhibit antigen-specific T cells and alleviate autoimmune myocarditis (40). In the *Brucella* infection model, Tip DCs exacerbate pathological damage, while IL-10 can alleviate liver damage by inhibiting their maturation (41). The above research results indicate that arginine metabolism is a key hub of immune function in DCs. The ARG pathway mainly mediates immune suppression and tissue repair, while the NOS pathway regulates immune response bidirectionally through NO production.

### Tryptophan

The effects of tryptophan on DCs are mainly mediated through its metabolic pathways and metabolites. The metabolism of tryptophan leads to the decrease of tryptophan content and the accumulation of tryptophan metabolites. Studies have shown that DCs differentiated under low-tryptophan conditions acquire a tolerogenic phenotype (42, 43) with increased expression of the inhibitory receptors immunoglobulin-like transcript 2 (ILT2), ILT3, and ILT4 (44). The metabolic pathways of tryptophan are mainly divided into three types: The Kynurenine (Kyn) pathway, the 5-hydroxytryptamine (5-HT) pathway, and the indole pathway. Of these, Kyn is the main metabolic pathway, and the key enzyme IDO1-mediated tryptophan degradation in this pathway is considered a key feedback mechanism for regulating the overactivity of immune responses (45).

Tryptophan is metabolized by indoleamine 2, 3-dioxygenase (IDO) into formylkynurenine, which is spontaneously converted into Kyn (46). IDO is a limiting enzyme in tryptophan metabolism, playing a key role in inducing tolerant DCs (47), Kyn produced by IDO catabolism can act on the aromatic hydrocarbon receptor (AhR) to inhibit the activation of the downstream NF-κB signaling pathway (48), and promote the secretion of the cytokines TGF-β and IL-10, which in turn promotes the differentiation of Treg (49). Tryptophan metabolism has long been suggested to be relevant in the pathophysiology of allergic disorders, including asthma. In allergen-specific immunotherapy for asthma, antigens attached to DCs induce reduced airway inflammation and inflammatory cytokines, enhanced tryptophan metabolism, and increased Tregs and IDO (50). A previous study showed that the DCs vaccine used to inhibit type 1 diabetes activated IDO1 expression *in vivo* by stimulating the nonclassical NF-κB pathway, which led to the suppression of DC-mediated type 1 diabetes autoimmunity (51). Chu et al. found that tyrosine kinase inhibitors (TKIs) imatinib and dasatinib can suppress the function of IDO in DCs and modify DCs to increase the activation of allogeneic T cells. Specifically, the suppressive effect of TKIs on tryptophan metabolism may be caused by blocking the c-Kit pathway in DCs. Overall, inhibiting IDO can be used to regulate DCs immunogenic activity and may potentially be applied in DC-based cancer immunotherapy (47). Arg1 and IDO1 are immunoregulatory enzymes catalyzing the degradation of L-arginine and L-tryptophan, respectively, resulting in local amino acid deprivation. Grohmann et al.’s study revealed for the first time that there is an interaction between the two in DCs: IDO1 phosphorylation and consequent activation of IDO1 signaling in DCs was strictly dependent on prior expression of Arg1 and Arg1-dependent production of polyamines. Their results demonstrate that arginine and tryptophan immunoregulatory pathways are functionally integrated in DCs (52). Further, it has been shown that the 5-HT pathway is able to regulate DCs migration, cytokine and chemokine release, and T cell initiation capacity *in vitro* and *in vivo* (53).

In addition to key metabolic pathways, tryptophan metabolites 3-hydroxyanthranilic acid (3-HAA) can also regulate DCs’ function. Lee et al. found that 3-HAA treatment significantly reduced the production of IL-12, IL-6, and TNF-α in bone marrow-derived DCs stimulated with LPS. Maturation markers CD40, CD80 and CD86 were also significantly reduced. Besides, 3-HAA treatment reduced the ability of DCs to stimulate T cell activation and differentiation both *in vitro* and *in vivo*. Finally, the authors observed that p-JNK and p-38 levels were reduced in 3-HAA-treated DC2.4 cells and bone marrow-derived DCs. This indicates that tryptophan metabolite suppresses T-cell responses by inhibiting DCs activation (54). Celiac disease is an autoimmune intestinal disease induced by the intake of gluten by genetically susceptible individuals. The patient’s immune system responds to certain gluten proteins, leading to excessive activation of immune cells such as DCs in the intestine and an increase in inflammation-related cytokines such as TNF-α and IL-12 (55). Studies have shown that tryptophan and its metabolites limit the expression of HLA-DR, CD83, and CD86, in addition to promoting the secretion of TGF-β and IL-10, which limits the secretion of IL-12 and TNF-α, to inhibit the inflammatory storm caused by gluten protein. This indicates that the overactivation of DCs’ immune function in patients with celiac disease can be rescued by tryptophan, highlighting their potential as therapeutic agents for celiac disease (56).

### Glutamate/glutamine

Glutamine is the most abundant amino acid in the blood and the main precursor of glutamate (57). Glutamine and glutamate can enter the tricarboxylic acid (TCA) cycle and play a role as necessary carbon sources in the synthesis of biomolecules and fatty acids. With the help of the transport system, extracellular L-glutamine crosses the plasma membrane and is converted to alpha-ketoglutarate (α-KG) through two pathways, glutaminase (GLS) I and II. On the contrary, α-KG can be converted into glutamine through glutamate dehydrogenase (GDH) and glutamine synthetase (GS), or into CO_2_ through the TCA cycle to provide energy to cells (58).

DCs expressed glutamate transporters, glutamate-specific vesicular transporters and were capable of fast glutamate release through a Ca^2+^-dependent mechanism (59). Glutamate receptors are widely present in immune cells such as T cells, B cells, DCs, and macrophages, making it possible for glutamate to play a role in both innate and adaptive immune systems (60, 61). T cells express a series of glutamate receptors on their surface, among which NMDA receptors (NMDARs) are glutamate-gated ion channels. The glutamate released by DCs binds to NMDA receptors on the surface of T cells, leading to the opening of Ca^2+^ channels, triggering of the sustained Ca^2+^ signal, and core crushing of T cells to thymocyte-DC contact zones (59, 62, 63). The glutamate released by DCs can bind to NMDAR, which can lead to focal responses in T cells under apoptosis (59). Although the TCA cycle involving glutamine is one of the main sources of energy in DCs, in some cases, DCs can develop immune tolerance to prevent hyperactivation and auto-immune diseases. In this case, the tolerance of DCs is maintained by the reduction of pyruvate and glutamine oxidation, as well as the interruption of the TCA cycle (64).

In addition to serving as a carbon source, glutamate is also an important nitrogen source for the synthesis of nucleotides, glucosamine, and other non-essential amino acids through transamination or deamination (65). Glutamine provides the necessary reduced nitrogen form for the synthesis of purine and pyrimidine bases (66). Similarly, the initial steps in pyrimidine biosynthesis involve the condensation of nitrogen derived from glutamine with bicarbonate and ATP to generate carbamoyl phosphate. Ultimately, additional glutamine is consumed during the synthesis of cytidine triphosphate (CTP) from uridine triphosphate (UTP) (67). In addition, glutamate is a key component in the synthesis of antioxidant-reduced glutathione (GSH). The GPX4-GSH antioxidant system plays a crucial role in combating oxidative stress and ferroptosis (68). Glutathione is a tripeptide synthesized from cysteine, glutamate, and glycine. In DCs, glutathione plays a critical role in maintaining REDOX homeostasis and protecting DCs from oxidative stress, lack of glutathione inhibits DCs maturation and the subsequent production of pro-inflammatory cytokines (69). Cystine/glutamate antiporter (Xc-system, xCT; ‘x’ indicates transporters for anionic amino acids; The subscript ‘C’ indicates that the transporter accepts cystine; The subscript ‘T’ indicates the transporter.) can transport cysteine into the cell in its oxidized form in exchange for glutamate and modulates the intracellular glutathione content and glutathione efflux from DCs. In this process, cystine/glutamate is transported, and glutathione homeostasis is regulated in DCs (70). xCT can promote the maturation of mononuclear-derived DCs, after inhibiting the xCT, the expression of MHC class I and II, CD80, and DC-SIGN on the surface of DCs is suppressed. However, the xCT does not affect LPS-induced DCs phenotype maturation and antigen uptake. Interestingly, xCT affects the antigen-presenting potential of DCs, and inhibition of xCT activity interferes with the DCs presentation of OVA to OT-II cells and suppresses the DC-stimulated proliferation of OT-I and OT-II T cells (71).

Given the impact of xCT on antigen-presenting potential of DCs, and the role of xCT in cancer exhaustively described by previous reports (including ferroptosis (72, 73), nutrient dependency (74), and cancer therapy (75)) (70), a new potential regulatory axis is proposed. The unfolded protein response (UPR) marker protein kinase RNA-like ER kinase (PERK), as a key sensor in the UPR, is activated when endoplasmic reticulum stress occurs and phosphorylates its downstream transcription factor ATF4, ATF4 directly binds upstream of the xCT promoter and promotes its transcription, ensuring sufficient cystine uptake and maintaining intracellular redox homeostasis. Whereas cancer cells with PERK-deficiency could induced ferroptosis and thus exert anti-cancer effects (76). In previous reports, Xing et al. innovatively proposed that tripartite motif protein family member 29 (TRIM29) inhibits type I interferon production by myeloid DCs in response to DNA and RNA viruses (77, 78). Their latest research shows that TRIM29 promotes SUMOylation of PERK to maintain stability (79). Given the role of PERK in xCT-mediated ferroptosis, could its upstream molecule, TRIM29, play other roles in DCs through PERK/xCT in addition to regulating type I interferon secretion? In addition, the role played by the TRIM29/PERK/ATF4/xCT axis in the antigen-presenting events of DCs deserves further validation and exploration.

Oxidative stress changes APC function, Glutathione can interfere with the release of DCs cytokines and regulate Th1 differentiation. The degradation of glutathione in DCs using a hiol-alkylating agent, diethyl maleate (DEM) can inhibit the expression of co-stimulatory molecules and Th1-polarizing cytokine IL-12 in DCs. In the DCs of delayed hypersensitivity (DTH) mouse model, the degradation of glutathione can significantly inhibit the release of Th1 cytokines IFN-γ and TNF-β, in contrast, the message level of a representative Th2 cytokine, IL-4, was unaffected by Glutathione. Besides, the expression of the important transcription factor Foxp3 in Treg is also not affected by glutathione. This indicates that glutathione depletion downregulates Th1 immunity through a perturbation of DCs maturation and IL-12 production (80). Similarly, a previous study has shown that intracellular glutathione redox status in DCs regulates the polarization of the Th1/Th2 balance. Raising endogenous GSH levels can increase LPS-induced IL-27 and IL12 production, thereby promoting Th1 response and inhibiting Th2. IL-27 siRNA can reverse this phenomenon, indicating that glutathione redox regulation of T-cell polarization depends on IL-27 production (69).

Nutrient and metabolite alterations occur in the TME and affect tumor-immune interactions (81). Glutamine has received widespread attention due to its metabolic role in cancer cells (82, 83). Guo, C. et al. identified glutamine-mediated intercellular metabolic crosstalk between tumor cells and cDC1s that support tumor immune evasion and revealed that the acquisition and signaling of glutamine in cDC1s are limiting events for DCs activation and hypothesized targets for cancer treatment (84). Chemotherapy can reduce the expression of glucose transporters and impair glucose uptake in cancer cells. In the case of decreased intracellular glucose in cancer cells, malate 2 (ME2) metabolizes glutamine-derived malic acid into pyruvic acid, thereby supplementing the Warburg effect. This provides a new perspective on the relationship between metabolism and chemotherapy resistance (85).

As mentioned earlier, Glutamate lies in the intersection of multiple metabolic pathways and participates in the amino acid metabolic cycle through transamination reactions (e.g., glutamine transaminase/glutamine transaminase, ALT/AST) to produce α-ketoglutarate (a TCA cycle intermediate), which connects carbon and nitrogen metabolism. Arginine metabolism releases glutamate-derived amino acids via the urea cycle. The kynurenine pathway of tryptophan generates kynurenine and NAD^+^, where NAD^+^ synthesis is dependent on amino acids supplied by glutamine metabolism. The metabolism of arginine, tryptophan and glutamate is closely linked within the cell through shared intermediary metabolites, signaling pathways and regulatory networks, and these three amino acids are synergistically involved in core life activities such as cellular energy metabolism, redox homeostasis, signaling, and immunomodulation (**Figure 3**).

### Alanine

Toll-like receptor 4 (TLR4) recognizes pathogen-associated molecular patterns (PAMPs) and initiates innate immune responses. Immature DCs express high levels of TLR4, which triggers a cascade of responses in the TLR4 pathway when DCs are stimulated by antigens, leading to DCs expressing high levels of chemokine receptors, maturing during migration and initiating an adaptive immune response (86, 87). However, previous reports showed that TLR-4 can also be activated by L-alanine. In a study of multidrug-resistant bacteria, exogenous L-alanine promoted the phagocytosis of pathogenic species and activated TLR-4 signaling by enhancing the biosynthesis and secretion of fatty acids (FAs), including palmitate. Mechanically, Palmitate enhances the binding of LPS to TLR4, thereby promoting TLR4 dimer formation and endocytosis for subsequent activation of the PI3K/Akt and NF-κB pathways and bacteria phagocytosis (88, 89). The mouse model also revealed that L-alanine metabolism and phagocytosis were strongly correlated with mouse survival. It suggests that modulation of the alanine metabolic environment is a plausible approach for combating multidrug-resistant bacteria infection (89). Coincidentally, a study by Xing et al. provided a new insight between alanine metabolism and the inflammation-related pathways. They found that poly(ADP-ribose) polymerase 9 (PARP9) recognizes and binds viral RNA, thereby recruiting and activating the PI3K/AKT3 pathway, independently of the mitochondrial antiviral-signaling pathway (MAVS). PI3K/AKT3 then activates IRF3 and IRF7, mediating type I IFN (IFN-I) production. Given that alanine metabolism is involved in the activation of the PI3K/Akt during bacterial phagocytosis, we believe that this process contributes, at least in part, to the production of IFN-I. Therefore, PARP9 potentially serving as a critical sensor and regulatory hub for alanine in activating the PI3K/Akt pathway and producing IFN-I during bacterial phagocytosis (90).

In addition to the facilitation of TLR4 signaling activation, alanine is also very useful to linker peptide for antigen presentation. Vaccines based on tumor-specific antigens are promising new therapeutic strategies, neoantigens can be presented to T cells by DCs system as a pool of short (8 to 11 amino acids) or long (15 or more residues) individual synthetic peptides or as unique long single molecule that concatenates the different neoantigens spaced by linker sequences (prepared as DNA, RNA or soluble protein). No clear consensus is found in the literature about which type of linker would be optimal, as a variety of linkers has been used so far, including the naturally flanking residues of the neoantigen. A recent study has shown that alanine-based linkers, especially AAA spacers, significantly improve the presentation of new antigens (91). In contrast to essential amino acids that require multi-step biosynthesis, alanine can be made from pyruvate by a single transamination. Several studies have shown that a high intake of extracellular alanine is required during T-cell activation and that alanine deficiency leads to metabolic and functional deficits (92, 93). However, more research is needed to determine whether alanine directly modulates DCs’ immune function.

### Cystine

Inside the cell, cystine is rapidly reduced to cysteine and functions both as an intracellular antioxidant and as the rate-limiting precursor for the biosynthesis of the major cellular antioxidant glutathione (70). According to a previous research report, xCT modulates the functional expression of IDO in human monocyte-derived DCs. Blocking antiporter can increase both IDO mRNA and IDO enzymatic activity and this correlated with impaired DCs presentation of exogenous antigen to T cells via MHC class II and class I pathway. Unlike in macrophages, the activity of IDO in DCs is not regulated by IFN-γ but determined by the level of redox, which controls the enzymatic activity of IDO by regulating protein synthesis rather than protecting it from proteasomal degradation (94). T cells do not have the ability to input cysteine and convert it into cysteine inside the cell (95). The uptake of cysteine by T cells depends on APCs such as DCs. DCs input cystine through the xCT, which converts it into cysteine inside the cell and pumps it out through ASC-neutral amino acid transporters. T cells also pump cysteine output from DCs through ASC to meet redox requirements (96). A study showed that MDSCs compete with APCs for extracellular cystine, and in the presence of MDSCs, APC release of cysteine was reduced, thereby limiting the extracellular pool of cysteine, and depriving T cells of the cysteine they require for activation and function (97).

### Histidine

Histidine is one of the essential amino acids, histidine decarboxylase (HDC) catalyses the production of histamine from histidine and is highly expressed in DCs. Histamine, as an important inflammatory mediator, regulates the immune activation or tolerogenic function of DCs by binding to histamine receptors (H1R, H2R, H3R, H4R) on the surface of DCs (98–101). The outer surface protein of *Bifidobacterium* contains active domains of cysteine- and histidine-dependent aminohydrolase/peptidase, which can activate DCs and trigger inflammatory responses. It is a novel microbial-related molecular model to actively participate in the cross-talk mechanisms between *bifidobacteria* and the host’s immune system (102). Mice overexpressing HDC will promote the formation of foam cells in atherosclerosis, HDC gene knockout mice, however, can inhibit the differentiation of M1 macrophages and DCs by activating the STAT6 signaling pathway due to the lack of histamine (103). iDC expresses two active histamine receptors, H1 and H2, and stimulation by LPS and simultaneous administration of exogenous histamine can act on H2 receptors for a short period of time, increasing IL-10 production and decreasing IL-12 secretion, and ultimately polarizing CD4^+^ T cells to a Th2 phenotype (100).

### Valine

Valine plays an important functional role in the metabolic health of mammals (104). The adverse effects of valine on metabolic homeostasis have been confirmed in obese animal or human subjects (105, 106). Valine treatment affects the intestinal microbiota and metabolite compositions and aggravates hepatic lipid deposition and adipogenesis (107). In advanced cirrhosis, there is impairment of the function and maturation of DCs, which has been shown to be related to an imbalance of extracellular branched-chain amino acids(BCAAs) (14). Depletion of extracellular valine inhibited DCs maturation, as the absence of valine in the medium inhibited the maturation of bone MoDCs, and the expression of the pDC activation marker CD83 was weak, whereas deprivation of leucine or isoleucine did not affect this process. BCAA, especially valine, modulated MoDC allostimulatory capacity and cytokine production. Elevating the extracellular valine concentration improved the allostimulatory capacity and IL-12 production dose-dependently in MoDCs from HCV cirrhotic patients (108).

With the latest development of metabolomic technology, studies in immune metabolism has flourished in the past few years, especially metabolic reprogramming, which is crucial for cell maintenance (109). Kim Arnold’s team presents a novel setup coupled to a secondary electrospray ionization-high resolution mass spectrometric (SESI-HRMS) platform to allow headspace analysis of immature and activated DCs in real time. Differences in metabolic characteristics between naive and activated DCs were identified, and pathway-enrichment analysis revealed three significantly altered pathways, including the TCA cycle, α-linolenic acid metabolism, and valine, leucine, and isoleucine degradation (110). This demonstrated that amino acid metabolic events, including valine, may have an important regulatory role in DCs activation.

### Leucine/isoleucine

The latest study suggests that leucine can regulate macrophage polarization and alleviate inflammatory cytokine storms through the mTORC1/LXRα signaling pathway (111). In DCs of the patients with cirrhosis, the lack of BCAAs including leucine can reduce the expression of phospho-S6 kinase, thereby inhibiting DCs maturation and promoting the malignant progression of cirrhosis. On the contrary, oral administration of BCAA granules to patients with advanced cirrhosis resulted in a significant increase in IFN-γ levels, which alleviated the deterioration of the disease caused by impaired DCs function, providing evidence for nutritional therapy with amino acids (14, 108). Leucine and glutamine are effective stimulators of mTORC1. The increased levels of leucine and enhanced glutaminolysis activate mTORC1 and subsequent c-Myc-mediated transcription of CD47. Treatment of tumor cells with leucine transporter L-amino acid transporter 2 (LAT2) inhibitors downregulated CD47 expression, thereby enhancing macrophage infiltration and phagocytosis of tumor cells (112). Circulating monocytes migrate from peripheral blood to tissues and can differentiate into DCs. Studies have shown that the ability of Mo in newborns to undergo apoptosis decreases after *E. coli* infection, leading to persistent inflammation. Supplementing with leucine can reduce persistent inflammation by promoting activation of the mTOR pathway (113). In a mouse model of food allergy, a high isoleucine diet exacerbated allergic reactions and increased the activity of sensitized DCs. In DCs, protein array analysis showed that the mTOR/AKT pathway mediates the function of isoleucine, and molecular docking suggested that Sestrin2 may be a potential receptor (114).

### Proline

DCs are classic innate immune cells, and studies have shown that proline modulates innate immunity in *Caenorhabditis elegans* by balancing redox homeostasis and activating the key transcription factor SKN-1 associated with pathogen defense (115). Proline is catalyzed by proline dehydrogenase enzyme (PRODH) to generate glutamate, which enters the TCA cycle to generate ATP and provides energy support for the activation of DCs and antigen presentation. Proline metabolism plays an active role in shaping the TME. For instance, to generate more collagen for the extracellular matrix (ECM), cancer-associated fibroblasts (CAFs) exhibit high proline synthesis rates (116). In addition, the increased proline synthesis in macrophages induced by early respiratory infections combined with allergen sensitization contributes to the occurrence of childhood allergic asthma (117).

### Phenylalanine

As early as 1964, Ryan and Carver scientists explained the mechanism by which phenylalanine inhibits the immune response to white throat toxins in rabbits and rats. It interferes with the uptake and intracellular mode of action of essential amino acids such as histidine, ornithine, and tyrosine to inhibit antibody synthesis and reduce immune response (118). Mature DCs highly express IL-4-induced gene 1 (IL4I1), which can secrete L-phenylalanine oxidase and promote the oxidative deamination of phenylalanine. This process affects the antigen presentation of DCs and their cross-talk with T-lymphocytes. Specifically, IL4I1-mediated deamination of phenylalanine temporarily downregulated TCR expression, inhibited CD4^+^ and CD8^+^T cell proliferation, and exerted a greater impact on memory T cells than naïve T cells. This suggests that the deamination process of phenylalanine may be a novel immune regulatory factor (119). In a liver metabolism-related study, the authors found that phenylalanine can be catalyzed to tyrosine, which is further oxidized to dopa and norepinephrine, promoting the proliferation of DCs (120). In patients with coronary heart disease, the upregulation of the serum phenylalanine to tyrosine ratio (Phe/Tyr) mediates higher levels of immune activation (121). In some diseases related to inflammation and the immune disorders, such as cancer, AIDS, and trauma, the concentration of phenylalanine in serum is significantly increased (122).

## Functions of gatekeepers: metabolic remolding and functional conversion on DCs

AATs are membrane-localized transport proteins that mediate the transfer of amino acids into cells or organelles. AATs have diverse functions in different microenvironments and are involved in a wide range of reactions from neurotransmission to acid-base homeostasis, intracellular energy metabolism and anabolic and catabolic reactions (123). DCs, as key APCs, are highly dependent on AATs for the regulation of intra- and extracellular metabolism. In recent years, AATs have been found to be a new target for immunotherapy by regulating the metabolic reprogramming and immune function of DCs.

### Alanine transporter

Solute carrier transporters (SLCs) mediate the cellular homeostasis of nutrients and metabolites, and up to 80% of small chemical molecules have been reported to be functionally dependent on SLCs (124). SLC1A4 can transport neutral amino acids such as alanine, serine, cysteine, and threonine (125). SLC38A2, a sodium-coupled neutral amino acid transporter, is highly expressed in DCs. In addition to transporting neutral amino acids, including alanine, it also mediates glutamine uptake (84). In pancreatic ductal adenocarcinoma (PDAC), pancreatic stellate cells (PSCs) compete with PDAC cells for alanine as a nutrient, PSCs utilize SLC1A4 to rapidly exchange and maintain environmental alanine concentrations. Moreover, PDAC cells upregulate SLC38A2 to supply their increased alanine demand. Cells lacking SLC38A2 fail to concentrate intracellular alanine and undergo a profound metabolic crisis resulting in markedly impaired tumor growth. This indicates that alanine transport mediated by SLC1A4 and SLC38A2 can regulate the metabolism, growth, and therapeutic resistance of PDAC by affecting PSCs (126). In view of the fact that SLC38A2 and SLC1A4 compete for alanine in pancreatic cancer, we speculated whether SLC38A2, which is highly expressed in DCs, also has a similar phenomenon in the immune microenvironment to meet its function.

### Arginine transporter

The cationic amino acid transporter protein SLC7A1 (CAT1) is an important L-Arg transporter protein for T cells (127), and SLC7A2 (CAT2) is highly expressed in DCs as an inducible L-Arg transporter actively involved in DCs activation and anti-inflammatory responses, which are essential for innate immunity. Upon activation by antigenic stimulation, DCs upregulate SLC7A2 or SLC6A14 (a cationic amino acid transporter) to meet arginine requirements (e.g. NO synthesis or immune regulation), and the activation of DCs is suppressed in SLC7A2-deficient mice (128). In *Mycobacterium bovis* or *Bacillus Calmette-Guérin* infected DCs, the expression of genes SLC7A2 is up-regulated, and the end products of arginine metabolism, NO, is significantly increased (129). NO derived from DCs suppresses IL-17 production by T cells (130). Another study indicates that SLC7A2-deficient mice had spontaneous inflammation in their lungs, and marked eosinophilia, associated with up-regulation of eotaxin-1, was present in the bronchoalveolar lavage fluid of 3-week-old SLC7A2-deficient mice. The eosinophilia was gradually replaced by neutrophilia in adult mice, while eotaxin-1 levels decreased and GRO-α levels increased, examination of DCs activation revealed increased DCs activation in the lungs of SLC7A2-deficient mice (131). Bone marrow-derived DCs infected with *Salmonella* enhance arginine uptake by upregulating the expression of SLC7A1 and SLC7A2 (132). In addition, in DCs activated by *Leishmania (L) amazonensis* amastigotes, there is a significant and coordinated up-regulation of L-arginine transporter and Arg2 transcripts (133). In addition, usually transporting large neutral amino acids, L-type amino acid transporters (LAT) may indirectly influence arginine metabolism and regulate cell activation status through coupling with the mTOR signaling pathway (134). As broad-spectrum neutral and cationic amino acid transporters such as SLC6A14, SLC7A7 or SLC7A1 can be induced to be expressed in immune microenvironment to mediate arginine uptake during DCs activation.

### Histidine transporter

SLC15 family members are proton-coupled oligopeptide transporters in vertebrates (135). Highly expressed and mainly localized in endosomes and lysosomes of DCs, SLC15A3 is responsible for the transport of histidine and oligopeptides to cytosol (136). SLC15A4 is another member of the POT family, also located in endosomes and lysosomes, and shares similar transport characteristics with SLC15A3 (137, 138). In pDCs, SLC15A4 is required for TLR7- and TLR9-mediated IFN-I productions, as the mutation of SLC15A4 indicates that AP-3, as well as the BLOC-1 and BLOC-2 Hermansky-Pudlak syndrome proteins are essential for pDC signaling through TLR7 and TLR9. However, these proteins are not necessary for TLR7 or TLR9 signaling in cDCs and thus comprise a membrane trafficking pathway uniquely required for endosomal TLR signaling in pDCs (139). In addition, SLC15A4 has been identified as a factor associated with susceptibility to systemic lupus erythematosus in mice, and miR-31-5p is a target gene that directly binds to SLC15A4. The expression of miR-31-5p was downregulated and negatively correlated with the overexpression of SLC15A4 in PBMCs of SLE patients (140), the absence of SLC15A4 results in pDCs being unable to produce IFN-I in response to the TLR7/9 ligand *in vivo*, to counteract the excessive production of IFN-I during the course of SLE. Baccala et al. demonstrate that pDCs and the IFN-I are critical contributors to the pathogenesis of lupus-like autoimmunity (141). After infectious or sterile stimuli, SLC15A4 in DCs is recruited to phagosomes, SLC15A4‐deficient DCs infected with *Salmonella typhimurium* show reduced caspase-1 cleavage and IL-1β production, this correlates with peripheral NLRC4 inflammasome assembly and increased autophagy. Overexpression of constitutively active mTORC1 rescues decreased IL-1β levels and caspase1 cleavage and restores perinuclear inflammasome positioning. This indicated that SLC15A4 is a novel link between inflammasome activation and phagocytosis by coupling phagosomal content sensing to mTORC1 signaling (142).

### Glutamate transporters

The function of SLC1A2 in the nervous system has been well elucidated, as it prevents excitotoxicity by clearing accumulated glutamate outside cells (143). Recently, emerging evidence suggests that SLC1A2 is associated with autoimmune diseases of the central nervous system and peripheral tissues. Gan et al. demonstrated that LPS-stimulated macrophages increase expression of SLC1A2 via NF-κB signaling. SLC1A2 is necessary for inflammatory macrophage polarization through sustaining mTORC1 activation. Mechanistically, lysosomal SLC1A2 mediates lysosomal glutamate and aspartate efflux to maintain V-ATPase activation, which sustains macropinocytosis and mTORC1 activation (144). While in DCs, the xCT was highly expressed, its role is to mediate the extracellular uptake of cystine and intracellular release of glutamate. In xCT-deficient mice, LPS-induced DCs maturation and inflammatory status were significantly reduced compared to wild-type mice, which may be related to changes in the amount of cysteine transferred into the cell or glutamate transferred out of the cell by xCT (145). xCT is located on the surface of DCs, acts as a molecular brake on efferocytosis. In diabetic mice, xCT inhibited the release of the TGF-β family member GDF15 from DCs, resulting in slow wound healing. Mechanistically, xCT-deficient DCs were dependent on aerobic glycolysis using glucose derived from glycogen stores for increased efferocytosis (146). In the immune microenvironment, glutamate affects the crosstalk between DCs and T cells by acting on different types of glutamate receptors, which we mentioned in the section on glutamate/glutamine earlier. Rodrigo et al. found that, during the maturation process, DCs release a large amount of glutamate upon contact with T cells, which is mediated by xCT expressed in DCs. Metabotropic glutamate receptor 1 (mGlu1R) is expressed in T cells, when stimulated by glutamate released by DCs in the microenvironment, mediates enhanced T cell proliferation and secretion of Th1 and proinflammatory cytokines. Indicated that glutamate is a key regulator of T cell activation during T cell-DC interactions (147).

### Cystine transporters

In addition to glutamate, the cysteine/glutamine transporter xCT is one of the transporters of cystine. xCT transports intracellular and extracellular cysteine, and glutamate regulates DCs differentiation and antigen presentation. Specifically, blocking antiporter activity interferes with DCs differentiation from monocyte precursors, but the antiporter activities are not required for LPS-induced phenotypic maturation. Furthermore, inhibiting antiporter uptake of cystine interferes with the presentation of exogenous antigen to MHCII-restricted T cells and blocks cross-presentation on MHCI (71). Cysteine is transported over the plasma membrane predominantly by the neutral amino acid transporters ASCT1 and ASCT2, whereas cystine is exclusively transported by the xCT (148, 149). In tumor cells, xCT promotes tumor growth, accompanied by suppression of anti-tumor immunity, and the xCT loss in tumor cells acts synergistically with the immunotherapeutic agent anti-CTLA-4. Mechanistically, xCT controls the transfer of cysteine and the maintenance of intracellular redox dynamics, and the deletion of xCT in tumor cells leads to defective cystine uptake, accumulation of reactive oxygen species, and impaired tumor growth. Similarly, T cell proliferation in culture was exquisitely dependent on xCT expression (150). xCT is well known for its function of maintaining intracellular and extracellular redox. In DCs, intervening in the redox perturbation caused by xCT strongly induces IDO expression and activity. While IDO-competent DCs promote long-term immune homoeostasis by limiting exaggerated inflammatory responses and directing regulatory T-cell effector function (151).

## Concluding remarks and future perspectives

As professional APCs, DCs sense danger signals through pattern recognition receptors and play a ‘sentinel’ role in initiating adaptive immune responses (152). The activation status of DCs is deeply coupled to their metabolic pattern shifts, when DCs switch from quiescence to maturation upon antigen stimulation, metabolic conversion is required to generate energy and macromolecules from nutrients to match functional demands. This metabolic conversion directly affects DCs’ survival, proliferation, differentiation, and effector functions. For example, glycolysis, oxidative phosphorylation (OXPHOS) and the TCA cycle are major biometabolic pathways, which take place in the cytoplasm and mitochondria, respectively (Site of glycolysis: cytoplasmic matrix; site of TCA cycle: mitochondrial matrix; site of OXPHOS: inner mitochondrial membrane). Heterogeneity in metabolic levels of DCs in different activation states, anabolism is preferred in immunogenic DCs to support the inflammatory phenotype and the secretion of numerous cytokines and chemokines. The organism’s mechanism of maintenance of stability in the host makes tolerogenic DCs more inclined to catabolism to maintain the tolerogenic phenotype (153). There is an active switch between mitochondrial metabolism and glycolysis in order to support the requirements of the various metabolisms for DCs to function. While glycolysis provides energy for rapid proliferation and effector functions, OXPHOS provides energy for long-term survival and memory cell formation. Thus, immature DCs depend on OXPHOS to maintain homeostasis, and mature DCs enhance glycolysis and glutamine metabolism to support antigen presentation and cytokine secretion.

Amino acid metabolism has important roles in the functional regulation of DCs. In the resting state, amino acid acquisition and excretion (or donate functional groups for other amino acids) in the DCs are in dynamic equilibrium. While, during activation, some amino acids are actively regulated within the DCs to meet the following functional requirements: increasing biosynthesis to drive the production of effector molecules (154); and, in addition to serving as substrates for the synthesis of proteins, amino acids can also modulate the production of effector molecules by activating pathways or participating in metabolic events, as we summarize in **Table 1** and **Figure 3**. Feeding different ATP-producing pathways; for example, glutamine/glutamate is a core metabolic fuel for DCs’ function, glutamine provides ATP and metabolic intermediates to DCs through conversion to glutamate and α-KG into the TCA cycle, and in TME, DCs compete with tumor cells for glutamine as a nutrient (84). And the BCAAs leucine, isoleucine, and valine provide the coenzyme A (CoA) derivatives acetyl-CoA and succinyl-CoA, which enter the TCA cycle (155). We speculate that the increased BCAA demand in activated DCs (156) feeds the TCA cycle, at least partially. Some amino acids are also actively regulated in order to support redox homeostasis and metabolic rewiring in DCs; cystine is translocated intracellularly by xCT and reduced to cysteine, which serves as a substrate for the antioxidant GSH (70). The arginine-based ARG1/iNOS metabolic pathway regulates T cell activation thresholds via DC-T communication, thereby determining the balance of immune response and tolerance (24, 157, 158). In addition, amino acids have a profound influence on the fate of immune and tumor cells in the TME. Inflammation triggered by tumor progression induces sustained high expression of IDO by DCs and tumor cells in the TME, leading to tryptophan depletion and accumulation of Kyn (159). Kyn activates the AhR pathway in DCs, which leads to the generation of tolerogenic DCs and induces Treg differentiation (160–162).

Immune cells are outstanding models for studying the metabolic function of cells (163). During the immune response, immune cells alter their metabolic activity (164), the metabolic reprogramming of DCs is essential for determining immunophenotypes and responses. We have summarized the potential roles of different amino acids and their transporters in DCs regulation. However, further research is needed to determine whether some amino acids, such as alanine and glutamate, can directly regulate DCs immune function or regulate T cells immune response through DCs, and the interactions between different amino acids in the DCs metabolic regulatory network are not yet clear. The extensive functionality of amino acids and the complexity of their transporters have posed challenges for subsequent research, which, if identified, will also reveal numerous targets for drug development in related diseases. Combining spatial metabolomics (e.g. MALDI-MSI) with scRNA-seq multi-omics enables the resolution of metabolic adaptation mechanisms of DCs in microenvironments such as lymphoid tissues and tumor/inflammatory foci. In the future, it is expected to develop traceable metabolic probes (e.g. ^13^C-labelled glutamine nanoparticles) to visualize the metabolism of DCs *in vivo*, and fully analyze the spatial and temporal dynamics of DCs metabolism. In addition, based on the metabolic characteristics of DCs in patients (such as glycolysis-dependent type/fatty acid oxidation type), stratified treatment strategies should be formulated to achieve personalized treatment.
